# Children with Behavioural Problems Misinterpret the Emotions and Intentions of Others

**DOI:** 10.1007/s10802-019-00594-7

**Published:** 2019-11-04

**Authors:** Amy E. Wells, Laura M. Hunnikin, Daniel P. Ash, Stephanie H. M. van Goozen

**Affiliations:** 1 School of Psychology, Cardiff University, Cardiff, Wales UK; 2 Department of Criminology and Criminal Justice, University of Northampton, Northampton, UK; 3 Department of Clinical Child and Adolescent Studies, Leiden University, Leiden, Netherlands; 4 School of Psychology, Cardiff University Centre for Human Developmental Science, Park Place, Cardiff, CF10 7AT UK

**Keywords:** Antisocial behaviour, Theory of mind, Emotion recognition, Intentionality

## Abstract

Research indicates that the misinterpretation of other’s emotions or intentions may lead to antisocial behaviour. This study investigated emotion and intention recognition in children with behavioural problems and examined their relationship and relations with behaviour problem severity. Participants were 7–11 year old children with behavioural problems (*n* = 93, mean age: 8.78, 82.8% male) who were taking part in an early intervention program and typically developing controls (*n* = 44, mean age: 9.82, 79.5% male). Participants completed emotion recognition and Theory of Mind tasks. Teachers and parents rated children’s emotional and behavioural problems. Children with behavioural problems showed impaired emotion and intention recognition. Emotion recognition and intention recognition were positively related and inversely associated with behavioural problem severity and, independently of one another, predicted behavioural problems. This study is the first to show that children with behavioural problems are impaired in identifying others’ emotions as well as intentions. These social cognitive processes were found to be related and inversely associated with severity of behavioural problems. This has important implications for intervention and prevention programmes for children with behavioural difficulties.

**Electronic supplementary material:**

The online version of this article (10.1007/s10802-019-00594-7) contains supplementary material, which is available to authorized users.

## Introduction

Antisocial behaviour (ASB) in children is an umbrella term that covers a broad range of behaviours that can be operationalised and studied in different ways, including psychiatric diagnoses of oppositional defiant disorder (ODD), conduct disorder (CD) and disruptive behaviour disorder (DBD). Behavioural problems in childhood are associated with subsequent ASB during adulthood (Sainsbury Centre for Mental Health 2009) and a diagnosis of CD in childhood is a criterion for a diagnosis of antisocial personality disorder (ASPD) in adulthood (Rhee and Waldman 2002). Common across diagnoses of ODD, CD and DBD are displays of chronic and repetitive aggressive behaviours, a disregard for the rights of others and a violation of social norms (American Psychiatric Association 2013). Dysfunctions in social cognition are hypothesised to play a causal role in the development of ASB. Social cognition can be defined as the processing of information which leads to the “accurate perception of the dispositions and intentions of other individuals” (Brothers 1990, p.28). As such, emotion recognition and Theory of Mind (ToM) are two key aspects of social cognition (Mier et al. 2010) and deficits in both have been implicated in the development of ASB.

Emotions serve a communicative function (Darwin 1871) and the accurate recognition of emotions is crucial for interpersonal interactions and social functioning (Herba and Phillips 2004). As such, an impairment in emotion recognition can have negative consequences, including displays of ASB (Blair 2003). Research has consistently shown that individuals who display ASB show an impairment in emotion recognition, specifically in the recognition of negative emotions. An impairment in facial emotion recognition ability has been reported in numerous antisocial populations, including antisocial children (Van Goozen 2015), adolescents with CD (Fairchild et al. 2009) and youth offenders (Bowen et al. 2014). Dysfunction of the amygdala is hypothesised to underlie the emotion recognition deficits observed in antisocial individuals (Marsh and Blair 2008). In healthy populations, the amygdala is activated in response to fearful stimuli (Whalen et al. 2001). However, structural imaging work has shown individuals with CD have amygdala abnormalities (Fairchild et al. 2011) and individuals with amygdala damage are impaired in recognising fear (Adolphs et al. 2005).

ToM refers to the ability to represent and understand the mental states of others (Hein and Singer 2008) and one of the main aspects of ToM is understanding the intentions of others (Mohammadzadeh et al. 2016). Mohammadzadeh et al. (2016) suggest that displays of ASB may be due to the misinterpretation of another’s intentions which then lead them to act inappropriately (i.e., aggressively). This may be reflective of a hostile attribution bias (HAB). A HAB refers to the tendency of antisocial individuals to attribute hostile intent to others in ambiguous situations (Nasby et al. 1980) and to consequently display inappropriate or aggressive behaviour. Indeed, studies have found low levels of ToM to be associated with more HABs (Choe et al. 2013).

However, Jones et al. (2010) found no difference in intention recognition between boys with behavioural problems and typically developing controls. In their study, Jones et al. (2010) used the Animated Shapes Task (AST; Abell et al. 2000; Castelli et al. 2002) as a measure of ToM. During the AST, participants watch video clips of animated triangles moving around and are asked to describe what is happening. As well as assessing participants’ understanding of the clips, the AST assesses participants’ ability to ascribe mental states and intentions to the triangles. Jones et al. (2010) found there was no difference in the ability to ascribe intentions to the triangles between children with behavioural problems and typically developing controls. However, the AST is a verbal task and previous research has consistently found ToM to be associated with language ability (Cutting and Dunn 1999); participants’ verbal ability was not controlled for in the study conducted by Jones et al. (2010).

Like emotion recognition, the amygdala has been hypothesised to contribute to ToM and the ability to correctly recognise and attribute intentions to others (e.g. Shaw et al. 2004; Skuse 2003). As such, studies have sought to establish whether these two aspects of social cognition are related. Mier et al. (2010) examined emotion recognition and the recognition of emotional intentions in typically developing individuals. They found a positive correlation between emotion recognition and intention recognition and overlapping brain activity during the emotion recognition and intention recognition tasks. They suggest that emotion recognition is a prerequisite for the recognition of emotional intentions as it preactivates the neuronal network necessary for intention recognition. Corden et al. (2006) explored the relationship between emotion recognition and performance on the AST. They found that around 10% of a sample of University staff and students showed low fear recognition (<50% accuracy) and those with low fear recognition ascribed significantly less correct intentions to the triangles. Thus, Corden et al. (2006) suggest that poor fear recognition is predictive of a pattern of social cognitive deficits.

Although research suggests that emotion recognition and intention recognition are related, to our knowledge this relationship has not yet been explored in children displaying behavioural problems. Coricelli’s (2005) model of ToM suggests it is a two component process comprised of (1) an unconscious, automatic process involving emotion recognition and (2) a conscious, voluntary process based on intentionality and empathy. Thus, if individuals are impaired in recognising emotions, as children with behavioural problems have consistently been shown to be (see Van Goozen 2015 for a review), we would expect them to also display an impairment in recognising others’ intentions.

The current study took place in the context of a broader project that aimed to reduce behaviour problems in children at-risk for future criminal behaviour through an early and targeted intervention. A unique initiative in Northamptonshire, The Early Intervention Hub, brings together police and social care and aims to intervene at an early stage when cases to not yet meet the threshold for help. In collaboration with researchers at Cardiff University, police community support officers (PCSOs) delivered the Cardiff Emotion Recognition Training (CERT; Hunnikin and Van Goozen 2018) to children who display an impairment in emotion recognition. Emotional skills are crucial for children’s life chance and developing these skills at an early age provides an opportunity to reduce ASB and violence (The Early Intervention Foundation 2018). In order for the CERT to be as effective as possible in improving emotion recognition ability and behaviour it is necessary to examine other areas of socio-emotional functioning, such as the ability to recognise other people’s intentions, in children displaying behavioural problems.

The current study compared emotion recognition ability and levels of ToM in children displaying behavioural problems to those of typically developing controls. In line with previous literature, we expected children with behavioural problems to show an impairment in emotion recognition, specifically the recognition of negative emotions. Moreover, given the proposed role of misunderstanding intentions in the development of ASB (Mohammadzadeh et al. 2016) we also expected children with behavioural problems to show a specific impairment in understanding intentionality. Given the previously reported relationship between emotion and intention recognition (Skuse 2003) we expected these constructs to be related, and hypothesised that difficulties in emotion and intention recognition would both be associated with severity of behaviour problems.

## Method

### Ethical Statement

All aspects of the research were approved by the Cardiff University School of Psychology Research Ethics Committee. Informed written consent was provided by the participant’s parents/guardians and informed written assent was obtained for the participants.

### Participants

137 children (112 male) aged 7–11 years old (*M* = 9.12, *SD* = 1.22) took part from schools across England and Wales. Participants were assigned to one of two groups: the behavioural problems group (BP) or the typically developing group (TD). Participants in the BP group were part of the Early Intervention Hub and were referred into the study by teachers, family support workers or PCSOs. The children in the Hub do not have a formal mental health diagnosis but already show behavioural and/or emotional problems. After referral to the study, the child’s teacher completed the Strengths and Difficulties Questionnaire (SDQ; Goodman 1997) to confirm behavioural status for the last 6 months. Children scoring in the ‘slightly raised’ or ‘above’ range for conduct and/or peer problems (≥ 3 out of 10) and/or ‘slightly lowered’ or ‘below’ range for prosocial behaviour (≤ 5 out of 10) were eligible to be included in the BP group. Raised scores on the SDQ peer problems subscale are reflective of the interpersonal problems individuals with behavioural problems display (Hunnikin and Van Goozen 2018) while lowered scores on the SDQ prosocial subscale provided an indirect measure of callous and unemotional (CU) traits (Blair et al. 2014; Kimonis et al. 2016). The hyperactivity subscale was not used as an eligibility criterion as emotion recognition impairments in children with disruptive behaviour appear to be specific to those with behavioural problems and are not part of the ADHD spectrum (Airdrie et al. 2018) and research suggests that ToM remains intact in individuals with ADHD (Charman et al. 2001). Children in the TD group were not part of the Hub and were referred by their teachers for not showing any behavioural or emotional problems and showed total SDQ scores in the ‘close to average’ range (teacher SDQ total score: ≤ 11 out of 40; parent SDQ total score: ≤ 13 out of 40).

### Materials

#### Demographic and Behavioural Characteristics

The two subset form of the Wechsler Abbreviated Scale of Intelligence (WASI; Wechsler 1999; vocabulary and matrix reasoning) was used to provide an estimated IQ score. The vocabulary subtest was used to provide an estimate of verbal IQ. Socioeconomic status (SES) was estimated using the Office for National Statistics estimates of average household total weekly income based on each participant’s postcode (Low = £0–£520; Middle = £521–£670; High = £671 + l; Hubble et al. 2015).

The SDQ (Goodman 1997) is a 25-item questionnaire that assess areas of emotional and behavioural difficulties and strengths. The SDQ is a widely used, valid and reliable measure (Stone and Otten 2010). The prosocial subscale was used as an indirect measure of CU traits given the DSM-5’s description of CD with CU traits as “with limited prosocial emotions” (Blair et al. 2014) and Kimonis et al. (2016) have shown significant correlations between the prosocial subscale of the SDQ and CU traits.

#### Facial Emotion Recognition

The Facial Emotion Recognition (FER; Bowen et al. 2014) test was used to measure children’s ability to recognise faces displaying happy, sad, fearful, angry and neutral expressions. Children viewed 60 faces on a laptop displaying one of the five expressions at either a low or a high intensity. Each face was presented alone for three seconds and then with the question “What emotion (if any) is this person showing?” with the five emotion labels presented in a numbered list either to the left or right of the face and participants were asked to indicate their response on the computer.

#### Theory of Mind

The Animated Shapes Task (AST; Abell et al. 2000; Castelli et al. 2002) was used to assess ToM. The task measures individuals’ ability to attribute mental state descriptions to animations of shapes interacting. The task consisted of six video clips of a big red triangle and a small blue triangle moving around on a framed white background. There were two conditions of animation: goal directed (GD) and ToM. In the GD clips interactions between the triangles did not involve mental states whereas in the ToM clips they did. Participants were shown the animations on a laptop and asked to describe what they thought was happening in each clip. Participants’ responses were recorded and later transcribed and scored according to the criteria given in Castelli et al. (2002). Responses were scored on three dimensions: (1) intentionality (0–5): participants’ ability to ascribe mental states to the triangles; (2) appropriateness (0–2): participants’ understanding of the clip; (3) length of answer (0–4): the number of clauses in an answer. Inter-rater reliability was 0.83 as confirmed between two blind coders using a subset of the data (15%).

### Statistical Analyses

Differences in demographic and behavioural characteristics between groups were analysed using independent samples t-tests for continuous variables and X^2^ tests for binary variables. Spearman’s rho correlations were used to examine relationships between behavioural characteristics and ToM and a multiple regression analysis was used to determine whether emotion and intention recognition, could independently of each other, predict behavioural problems. Percent correct for total emotion recognition was calculated by taking the mean score of happy, sad, fear, anger and neutral recognition and percent correct for negative emotions was calculated by taking the mean score of sad, fear and anger recognition. Multivariate analyses of covariance (MANCOVA) were run to determine any between group differences for emotion recognition and for intentionality and appropriateness scores on the AST. Bonferroni corrections were used to adjust for multiple comparisons.

### Covariates

Due to the verbal nature of the FER and AST tasks and prior research that shows an association between verbal ability and emotional intelligence (Hogan et al. 2010), on the one hand, and ToM (Cutting and Dunn 1999), on the other, verbal IQ was included as a covariate in the analyses for both tasks, and length of answer was included as a covariate for analyses pertaining to the AST. IQ, age and SES were not included as covariates in the analyses. SES was not significantly correlated with performance on either the FER or AST (*p* < 0.05). IQ was significantly correlated with performance on the FER but not with performance on the AST. A hierarchical regression analysis showed that FER significantly predicted behavioural problems even when controlling for IQ. Age was significantly correlated with performance on the AST, but not with performance on the FER. A hierarchical regression analysis showed that while performance on the AST significantly predicted behavioural problems, age did not. As FER and AST predicted behavioural problems even when controlling for IQ and age respectively these demographic variables were not included as covariates in the analyses.

## Results

### Demographic and Behavioural Data

Participants in the TD group were significantly older, had a higher SES, IQ and verbal IQ than the BP group (see Table 1). Age was significantly correlated with performance on the AST, *r*(135) = 0.22, *p* = 0.009, but not with performance on the FER, and IQ was significantly correlated with performance on the FER, *r*(135) = 0.33, *p* < 0.001, but not with performance on the AST. See Supplement 1 for a table detailing the relationship between all the demographic variables and outcome measures. The groups were matched on gender and there was no difference in emotion recognition by gender, *t*(135) = −0.16, *p* = 0.877, nor was there an effect of gender performance on the AST, *t*(135) = 0.63, *p* = 0.530. Participants in the TD group exhibited significantly fewer conduct and peer problems, had a lower total SDQ score and higher prosocial score than the BP group.

**Table 1:** Demographic and behavioural characteristics of participants

	TD	BP	*p* value
Age (years)	9.82 (1.12)	8.78 (1.14)	<0.001
IQ	103.34 (17.45)	92.40 (13.32)	<0.001
Verbal IQ	52.68 (10.91)	45.18 (10.57)	<0.001
Gender			0.646
% Male	79.5	82.8	
% Female	20.5	17.2	
SES			<0.001
% Low	0	7.9	
% Medium	18.4	53.9	
% High	81.6	38.2	
SDQ score			
Total	7.22 (3.03)	17.92 (6.27)	<0.001
Conduct problems	1.05 (1.05)	4.12 (2.63)	<0.001
Peer problems	1.71 (1.38)	3.68 (2.38)	<0.001
Prosocial behaviour	7.49 (3.15)	4.48 (2.8)	<0.001

Means are presented with standard deviations in brackets

*IQ* intelligence quotient (two-subtest WASI), *SES* socioeconomic status, *SDQ* Strengths and Difficulties Questionnaire, *TD* typically developing group, *BP* behavioural problem group

### Emotion Recognition

**Fig. 1:**
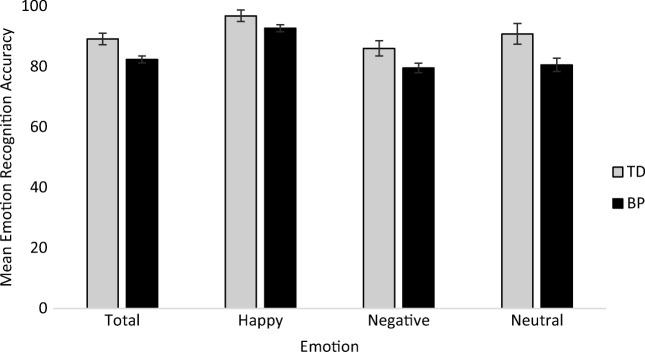
Estimated marginal means of total, happy, negative and neutral emotion recognition scores. Error bars are set at ±1 standard error. * = *p* < 0.05. TD = typically developing group, BP = behavioural problem group

BP participants scored significantly lower than TD participants for total, *F*(1, 128) = 8.43, *p* = 0.004, n_p_^2^ = 0.06, 95% CI [−11.39,−2.16], negative, *F*(1, 128) = 4.66, *p* = 0.033, n_p_^2^ = 0.04, 95% CI [−12.48,−0.544] and neutral recognition, *F*(1, 128) = 6.11, *p* = 0.015, n_p_^2^ = 0.05, 95% CI [−18.46,−2.05]. There was no difference between BP and TD participants for happy recognition, *F*(1, 128) = 3.04, *p* = 0.084, n_p_^2^ = 0.02, 95% CI [−8.71, 0.55] (see Fig. 1).

### Theory of Mind

BP participants scored significantly lower than TD participants on intentionality for ToM clips, *F*(1, 128) = 4.72, *p* = 0.032, n_p_^2^ = 0.04. There was no difference between BP and TD participants on intentionality for GD clips or appropriateness for either GD or ToM clips (see Table 2).

**Table 2:** Participant scores on the AST for the two groups of participants

	TD	BP	*p* value	Partial n^2^	95% CI
Intentionality					
GD (/10)	5.12	5.59	0.202	0.013	−0.20, 0.94
ToM (/20)	14.07	13.02	0.032	0.036	−2.01, −0.09
Appropriateness					
GD (/4)	3.45	3.19	0.127	0.019	−0.61, 0.08
ToM (/8)	4.05	3.49	0.090	0.023	−1.21, 0.09

Estimated marginal means are presented for intentionality and appropriateness subscales of the AST for the two conditions of film clip

*GD* goal directed, *ToM* Theory of Mind, *TD* typically developing group, *BP* behavioural problems group

### The Relationship Between Behavioural Problems, Emotion Recognition and Intentionality

Emotion recognition was significantly inversely related with conduct, *r*(130) = −0.21, *p* = 0.014, peer problems, *r*(130) = −0.28, *p* = 0.001, and total SDQ score, *r*(130) = −0.39, *p* < 0.001, while intentionality scores for ToM clips was inversely associated with conduct problems, *r*(130) = −0.20, *p* = 0.021, and total SDQ score only, *r*(130) = −0.25, *p* = 0.004. The ability to recognise others’ emotions and others’ intentions were significantly related, *r*(130) = 0.20, *p* = 0.019 and there was no relationship between prosocial behaviour and emotion recognition, *r*(130) = 0.08, *p* = 0.349, nor intentionality scores for ToM clips, *r*(130) = 0.10, *p* = 0.234 (see Table 3).

**Table 3:** Relationship between behavioural problems, emotion recognition and intentionality

	SDQ				FER	Intentionality	
	Conduct	Peer	Prosocial	Total	Total	GD	ToM
Conduct	–						
Peer	0.21*	–					
Prosocial	−0.47**	−0.23**	–				
Total	0.74**	0.63**	−0.44**	**–**			
Total FER	−0.21**	−0.28**	0.08	−0.39**	–		
GD intentionality	−0.04	−0.11	0.07	−0.04	0.01	–	
ToM intentionality	−0.20*	–0.12	0.10	0.25**	0.20**	0.43**	–

Values represent Spearman’s rho. Correlations *n* = 132. * = correlation is significant at the 0.05 level, ** = correlations is significant at the 0.01 level

*GD* goal directed, *ToM* Theory of Mind

Multiple regression analysis was used to determine whether emotion recognition and intention recognition (for ToM clips) predicted behavioural problems (as indicated by total SDQ score). The multiple regression model significantly predicted total SDQ score, *F*(2,130) = 14.80, *p* < 0.001, adjusted R^2^ = 0.17. Both emotion recognition and intention recognition significantly added to the prediction, *p* < 0.05 (see Table 4).

**Table 4:** Summary of multiple regression analysis

	B	SE_B_	β
Intercept	39.24	4.57	
Emotion recognition	−0.22	0.05	−0.36*
ToM intentionality	−0.45	0.21	−0.17*

B = unstandardized regression coefficient, SE_B_ = standard error of the coefficient, β = standardized coefficient, * = *p* < 0.05

## Discussion

The first aim of the current study was to assess and compare emotion recognition and intention recognition and attribution in children with behavioural problems and controls. Our results confirm previous findings of emotion recognition impairments in children with behavioural problems. However, for the first time, our results also indicate that children with behavioural problems are impaired in recognising other people’s intentions. Specifically, children in the BP group were found to be impaired in ascribing intentions and mental states to the triangles for ToM clips, but showed no impairment in their understanding of the clips or in ascribing intentions and mental states to the triangles for GD clips. However, in addition to differing in behavioural problems, the BP and TD groups differed on demographic variables and although unlikely to have influenced the results this needs to be taken into account when considering the findings of the current study. The second aim of the study was to explore relationships between emotion recognition, intention recognition and behavioural characteristics. We found that emotion and intention recognition were positively related and were inversely associated with behavioural problems and these two cognitive skills, independently of each other, predicted behavioural problems.

The results of the current study are in contrast to the findings of the only other study to look at intentionality in children with behavioural problems using the AST. Jones et al. (2010) found no significant difference in performance on the AST between participants with behavioural problems and a control group. There are a number of potential explanations that could account for the contrasting findings between our study and the one conducted by Jones et al. (2010). In the Jones et al. (2010) study the mean age of participants with conduct problems was 12 years while the mean age of children in the CP group in the current study was 8.86 years old. Schwenck et al. (2012) also used the AST and compared the appropriateness scores of children and young people with CD with the appropriateness scores of typically developing control group. In their work Schwenck et al. (2012) divided their sample into ‘young’ and ‘old’ (below and above 11.9 years old respectively) and found that older children described GD and ToM clips significantly more accurately. The difference in age between the participants in our study and the study conducted by Jones et al. (2010) could contribute to the difference in results. As previously mentioned, the BP and TD groups in the current study did differ in age and IQ so it is possible that this may account for the different findings between the current study and work by Jones et al. (2010). It is also of note that the sample size in the current study is much larger than the sample size used by Jones et al. (2010), who acknowledged that the modest sample size of their groups might mean they may have missed effects of a smaller magnitude.

Mohammadeszadeh et al. (2016) suggest that displays of ASB in individuals with behaviour problems may be due to their misinterpretation of another’s intention which then leads them to act inappropriately. The specific impairment in intentionality displayed by the BP group in the current study provides support for Mohammadzadeh et al.’s (2016) suggestion and is line with research that has found associations between impaired ToM and hostile attribution biases (HAB; Choe et al. 2013). In antisocial individuals a HAB has been shown to extend from interpreting ambiguous situations as hostile to interpreting neutral faces as angry (Leist and Dadds 2009). The association between emotion and intention recognition in the current study and results of previous work indicating a relationship between emotion and intention recognition (Corden et al. 2006; Mier et al. 2010) suggest that the misinterpretation of other people’s emotions and intentions may contribute to the development of a HAB which consequently may lead to aggressive, antisocial behaviours. Although the current study showed that emotion recognition and intention recognition are related processes, they independently of one another predicted behavioural problems. This highlights the need to consider these two aspects of social cognition separately and the role they play in the development of ASB when determining intervention and treatment plans for individuals with behavioural problems.

The current study found an inverse association between emotion recognition ability and the severity of both conduct and peer problems. Work conducted by Hubble et al. (2015) further illustrates the relationship between emotion recognition and the severity of conduct problems. Hubble et al. (2015) showed that fear, sadness and anger recognition can be improved in youth offenders following emotion recognition training and the improvement was associated with a significant reduction in the severity of crimes committed six months later. Previous research has shown that good emotion recognition enables individuals to initiate and maintain social relationships (Hunnikin and Van Goozen 2018) and the negative association between emotion recognition and severity of peer problems in the current study demonstrates that the reverse is also true.

Like emotion recognition, intention recognition was inversely associated with conduct problem severity. This is line with previous research showing that lower levels of ToM in childhood are associated with more externalising problems (Hughes et al. 1998). However, Hughes et al. (2000) found that ToM in ‘hard to manage’ pre-schoolers was not related to negative aspects of social interaction such as hurting a peer. The contrasting results may be due to methodological factors. In the study conducted by Hughes et al. (2000) behaviour was assessed by observing twenty minutes of play between the participant and their best friend. This is in contrast to the results of the current study and previous work that has found an association between aspects of ToM and conduct problems when using questionnaire ratings of behaviour.

CU traits are patterns of callousness, uncaring behaviour, reduced guilt and reduced empathy (Blair et al. 2014) and are displayed by a subgroup of children with conduct problems. There is some evidence to suggest impairments in social cognition in antisocial populations are dependent on the presence of CU traits and with individuals high in CU traits showing impairments in both cognitive empathy, which is effectively ToM (Blair 2008), and affective empathy (Anastassiou-Hadjicharalambous and Warden 2008). However, in the current study the prosocial subscale of the SDQ, an indirect measure of CU traits (Kimonis et al. 2016), was not found to be related with either emotion or intention recognition.

Although the current findings offer promising new avenues for research it is important to acknowledge the limitations of this study. Previous work has consistently found individuals with autism spectrum disorder (ASD) to show an impairment in ToM. Although in the current study participants were referred due to displays of disruptive behaviour and/or a lack of prosocial behaviour, there was no measure of ASD traits so we cannot rule out a contribution of ASD traits to our findings. Similarly, there was no direct measure of CU traits and previous research has shown deficits in social cognition can be dependent on the presence of CU traits (e.g. Jones et al. 2010). However, the prosocial subscale of the SDQ was used as an indirect measure of CU traits. As previously mentioned there were differences between the BP and TD groups in terms of demographic characteristics and as such we cannot rule out their contribution to our findings. However, hierarchical regression analyses found that emotion recognition and ToM predicted behavioural problems even when controlling for demographic characteristics suggesting it is unlikely that demographical differences are responsible for the difference between BP and TD groups in emotion and intention recognition.

Future research should aim to investigate ToM in children and young people displaying behavioural problems across different ages. Previous work has shown that symptoms of behavioural problems change with age (Brocki and Bohlin 2006) and as such, children’s developmental age may influence the relationship between behavioural problems and aspects of social cognition. Similarly, Dadds et al. (2009) showed that deficits in cognitive empathy associated with psychopathic traits were greatest in children and decreased with age. This suggests that a ‘catch in’ in cognitive empathy occurs over time in individuals high in psychopathic traits. It would be interesting to see whether a similar ‘catch up’ in intention recognition also occurs. Future studies should ensure participant groups are age matched to determine whether any differences in social cognition are attributable to behavioural problems, and not due to differences in age. In addition, future research should explore the relationship between emotion and intention recognition in children and young people with a diagnosis of CD and in other neurodevelopmental disorders characterised by externalising problems, such as attention deficit hyperactivity disorder (ADHD), to determine whether the same results are found as in the current study.

## Conclusions

In conclusion, the current study compared emotion recognition and intention recognition and attribution in children who were identified by their teachers as having behavioural problems to a group of typically developing controls, and also examined the relationship between emotion recognition, intention recognition and severity of behaviour problems. Our results indicate that children with behavioural problems show an impairment in both emotion and intention recognition and that these two components of social cognition are related. In addition, emotion and intention recognition were both inversely associated with severity of behavioural problems and independently of one another predicted behavioural problems. Our findings indicate that the well-documented deficit in emotion recognition in those with behavioural problems extends to the recognition and interpretation of the intentions of others. The possibility of a wider impairment in social cognition should be taken into account when considering the best intervention and prevention strategies for children who exhibit antisocial behaviour.

## Electronic supplementary material

ESM 1 (DOCX 12 kb)

## Abbreviations

FER: facial emotion recognition
HAB: hostile attribution bias
