# Supplementary material for: Children with Behavioural Problems Misinterpret the Emotions and Intentions of Others
Source: J Abnorm Child Psychol. 2019 Nov 4;48(2):213–21. doi: 10.1007/s10802-019-00594-7 (PMC6969861; doi:10.1007/s10802-019-00594-7)
Supplement: Supplementary file 1 — (DOCX 12 kb) [file 10802_2019_594_MOESM1_ESM.docx]

**Table 1**: Correlations between demographics and outcome variables for whole sample (n = 137)

|  | **FER** | **AST: Intentionality** |
| --- | --- | --- |
| **IQ** | .335** | .113 |
| **Verbal IQ** | .266** | .145 |
| **Age** | .146 | .280** |
| **SES** | .135 | .029 |

Notes: IQ = intelligence quotient (two subset-WASI), verbal IQ = WASI vocabulary subtest, SES = socioeconomic status, FER = facial emotion recognition, AST = animated shapes task. * = p < .05, ** = p < .01
